# Genomic Insight into the Spread of Meropenem-Resistant *Streptococcus pneumoniae* Spain^23F^-ST81, Taiwan

**DOI:** 10.3201/eid2604.190717

**Published:** 2020-04

**Authors:** Yi-Yin Chen, Yu-Chia Hsieh, Yu-Nong Gong, Wei-Chao Liao, Shiao-Wen Li, Ian Yi-Feng Chang, Tzu-Lung Lin, Ching-Tai Huang, Cheng-Hsiu Chiu, Tsu-Lan Wu, Lin-Hui Su, Ting-Hsuan Li, Ya-Yu Huang

**Affiliations:** Chang Gung Children’s Hospital, Taoyuan, Taiwan (Y.-Y. Chen, Y.-C. Hsieh, C.-H. Chiu, T.-H. Li, Y.-Y. Huang);; Chang Gung University, Taoyuan (Y.-N. Gong, W.-C. Liao, S.-W. Li, I.Y.-F. Chang, T.-L. Lin, T.-L. Wu, L.-H. Su);; Linkou Chang Gung Memorial Hospital, Taoyuan (Y.-N. Gong, W.-C. Liao, C.-T. Huang, T.-L. Wu, L.-H. Su)

**Keywords:** Streptococcus pneumoniae, 15B/C, invasive pneumococcal disease, PCV-13, meropenem resistance, genome sequencing, meningitis, pneumonia, sepsis, antimicrobial resistance, bacteria, AMR, Taiwan

## Abstract

Incidence of invasive pneumococcal disease caused by antimicrobial-resistant *Streptococcus pneumoniae* types not included in pneumococcal conjugate vaccines has increased, including a penicillin- and meropenem-resistant serotype 15A-ST63 clone in Japan. During 2013–2017, we collected 206 invasive pneumococcal isolates in Taiwan for penicillin and meropenem susceptibility testing. We found serotypes 15B/C-ST83 and 15A-ST63 were the most prevalent penicillin- and meropenem-resistant clones. A transformation study confirmed that penicillin-binding protein (PBP) 2b was the primary meropenem resistance determinant, and PBP1a was essential for high-level resistance. The rate of serotype 15B/C-ST83 increased during the study. All 15B/C-ST83 isolates showed an *ermB* macrolide resistance genotype. Prediction analysis of recombination sites revealed 12 recombination regions in 15B/C-ST83 compared with the *S. pneumoniae* Spain^23F^-ST81 genome. Pneumococcal clones rapidly recombine to acquire survival advantages and undergo local expansion under the selective pressure exerted by vaccines and antimicrobial drugs. The spread of 15B/C-ST83 is alarming for countries with high antimicrobial pressure.

*Streptococcus pneumoniae* is a major human pathogen that causes bacteremia, meningitis, pneumonia, and sepsis (*1*). Although the use of pneumococcal conjugate vaccines (PCVs) has dramatically decreased incidence rates of diseases caused by vaccine-targeted serotypes in children (*2*), the rates of pneumococcal disease caused by non-PCV serotypes have risen, including as 35B in the United States; 8, 12F, and 9N in the United Kingdom; 24F in France; 12F in Israel; and 15A in Japan (*3*,*4*). Of note, 35B and 15A have high-level β-lactam resistance (*3*,*4*).

Taiwan launched a national vaccination catch-up program in 2013, in which 1 dose of 13-valent PCV (PCV13) was administered to children 24–60 months of age. In 2014, the program was expanded to include 2 doses at 12–23 months of age, and in 2015, a 2+1 national infant immunization program was implemented (*5*). Since 2015, PCV13 coverage has been >90%, and the incidence of invasive pneumococcal disease (IPD) in children <5 years of age was reduced by 70%, from 18.9/100,000 children during 2010–2012 to 6.3/100,000 children during 2015–2017 (*6*). Surveillance data from the Taiwan Centers for Disease Control (Taiwan CDC) demonstrated that serogroup 15 isolates caused most IPD in children <5 years of age during 2015–2017, which increased from 0.67/100,000 children in 2010 to 2.61/100,000 children in 2017 (*6*). In Japan, serotype 15A sequence type (ST) 63 (15A-ST63) was highly associated with resistance to penicillin (MIC >2 mg/L) and meropenem (MIC >0.5 mg/L) (*3*). 

Meropenem is a broad-spectrum carbapenem antimicrobial drug recommended for initial empirical therapy in some countries and is indicated for treating bacterial meningitis caused by *S. pneumoniae* in children >3 months of age (*7*,*8*). Meropenem resistance seen in the *S. pneumoniae* 15A-ST63 clone in Japan was thought to be due to acquisition of penicillin-binding protein (PBP) 1a (type 13) via recombination with a formerly predominant global serotype 19A-ST320 vaccine strain (*3*). 

Before widespread use of PCVs in Taiwan, the Pneumococcal Molecular Epidemiology Network (PMEN) standardized nomenclature of prevalent multidrug-resistant international clones causing most invasive diseases, including Spain^23F^-ST81 (PMEN1), Taiwan^19F^-ST236 (PMEN14), Taiwan^23F^-ST242 (PMEN15), and 19A-ST320 (*9*,*10*). These clones usually harbored PBPs that are not susceptible to penicillins, third-generation cephalosporins, and meropenem (*3*,*11*). Because *S. pneumoniae* is highly recombinogenic, continued circulation of these highly β-lactam–resistant PBP determinants is of concern in the era of PCV13. A previous study in Taiwan demonstrated that the prevalence of non-PCV13 serotypes 15A-ST63, 15B-ST83, a single locus variant of Spain^23F^-ST81; and 23A-ST338 has increased since 2012 (*12*). 

We examined the in vitro activity of penicillin and meropenem against invasive pneumococcal strains isolated in Taiwan during 2013–2017, analyzed their genetic relatedness, and assessed the role of amino acid changes in PBP1a, 2b, and 2x in meropenem resistance. In addition, we used whole-genome sequencing (WGS) to characterize the prevalent clone and its relationship to historical strains.

## Materials and Methods

### Hospitals and Patients

During January 2013–December 2017, we collected invasive pneumococcal isolates at a 3,700-bed branch of Chang Gung Memorial Hospital (CGMH) in Linkou, northern Taiwan, and at a 2,700-bed CGMH branch in Kaohsiung, southern Taiwan. Since 2006, CGMH has archived all pneumococcal isolates from patients with IPD, which was defined as an illness in which *S. pneumoniae* was isolated from >1 normally sterile site. In addition, Taiwan has an active surveillance system that made IPD a nationally notifiable disease in 2007. Therefore, doctors must report clinical information and submit pneumococcal isolates to the Taiwan CDC (*6*). This study was approved by the research ethics committee of CGMH, Taiwan (approval no. 201801433B0).

### Bacterial Strains

We grew 206 nonrepetitive *S. pneumoniae* isolates at 37°C in Todd Hewitt broth supplemented with 0.5% yeast extract or on blood agar with 5% defibrinated sheep blood (Becton Dickinson, https://www.bd.com) in an atmosphere of 5% CO_2_. We used standard latex agglutination and quellung reactions for serotyping and the broth microdilution method to determine MICs of meropenem and penicillin for *S. pneumoniae* isolates (*13*). For penicillin, we defined the criteria for meningitis as MIC <0.06 mg/L, susceptible; and MIC >0.12 mg/L, resistant. We defined criteria for nonmeningitis as MIC <2 mg/L, susceptible; MIC = 4 mg/L, intermediate; and MIC >8 mg/L, resistant. For meropenem, we defined MIC <0.25 mg/L, susceptible; MIC = 0.5 mg/L, intermediate; and MIC >1.0 mg/L, resistant. We used the *S. pneumoniae* MLST database (https://pubmlst.org/spneumoniae) to perform multilocus sequence typing (MLST) by PCR for the 125 meropenem-nonsusceptible isolates identified from the 206 isolates (*14*).

### PBP Profiles

We performed PCR to compare the sequences of the transpeptidase regions of the *pbp1a*, *pbp2b*, and *pbp2x* genes of all 125 meropenem-nonsusceptible isolates by using 12 primers (Appendix 1 Table 1). We separated the resulting PCR products by using 1% agarose gel electrophoresis and extracted DNA for Sanger sequencing. We used BLAST 2.10.0 (https://blast.ncbi.nlm.nih.gov/Blast.cgi) to compare the nucleotide sequences of each PBP transpeptidase region to the PBP sequences in the US Centers for Disease Control and Prevention database (*15*) and in previous studies (*3*,*11*,*16*,*17*).

### Transformation of *S. pneumoniae*

We used the nonencapsulated laboratory strain R6, a meropenem-susceptible pneumococcus (MIC 0.015 mg/L), as the recipient in transformation studies. We used PCR to amplify the entire *pbp1a*, *pbp2b*, and *pbp2x* genes of the 15A-ST63 clinical isolates (Appendix 1 Table 1). We cloned the products into pJET1.2/blunt (ThermoFisher, https://www.thermofisher.com) according to the manufacturer’s instructions and transformed it to *Escherichia coli* DH5α. We extracted the *pbp* plasmids from DH5α cells with the Plasmid DNA Mini Kit (QIAGEN, https://www.qaigen.com) and then transformed it to *S. pneumoniae*, as described previously (*18*). We spread the transformation on Mueller-Hinton agar containing 5% sheep blood and different concentrations of meropenem. After a 24-h incubation, we selected transformants from the plates containing the highest meropenem concentration with colonies.

### WGS

To investigate evolutionary relationships of *S. pneumoniae*, we performed WGS on 27 isolates, including 24 meropenem-nonsusceptible isolates of serotype 15B/C-ST83 collected during 2013–2017 and 3 clinical isolates of serotype 23F-ST81 identified in our previous study (*10*). We used a QIAamp DNA Mini Kit (QIAGEN) to extract *S. pneumoniae* genomic DNA and prepared libraries for WGS sequencing by using the TruSeq Nano DNA High Throughput Library Prep Kit (Illumina, https://www.illimina.com). We multiplexed library samples and sequenced on an Illumina MiSeq with 2×300-bp paired-end reads. We used SPAdes version 3.13.0 (*19*) to quality trim raw reads before assembling by using k-mer values 21–77 in careful mode. We used QUAST (*20*) to evaluate this assembly, which produced an average of 140 contigs with an N50 length in 101,632bp. Because *S. pneumoniae* PMEN1 ATCC-700669 (GenBank accession no. FM211187), a serotype of the 23F-ST81 strain, is genetically related to ST83, a single locus variant of ST81, we chose this strain as a reference sequence for mapping. We deposited WGS data, ≈1.4 million pair-end reads per sample, to the Sequence Read Archive database (SRA; http://www.ncbi.nlm.nih.gov/sra) under accession nos. SRR8867342–68 (Appendix 1 Table 2). 

### Predicting Recombination Sites by Phylogenetic Analysis

We aligned the trimmed reads for the 27 isolates against the reference genome of ATCC-700669 by using Snippy version 4.4.5 (*21*), which uses the Burrow-Wheeler Aligner (https://github.com/lh3/bwa) to map the reads to the reference and then calls the subsequent single-nucleotide polymorphisms (SNPs) and insertions/deletions with FreeBayes per Garrison et al. (https://arxiv.org/abs/1207.3907). We used the whole-genome core SNPs alignment output from Snippy for downstream phylogenetic analysis, assessed recombination sites by using Gubbins version 2.3.4 (*22*), and generated a maximum-likelihood tree by using RAxML version 8.2.10 (https://github.com/stamatak/standard-RAxML). We ran RAxML by using the GTRCAT model, no rate heterogeneity, no ascertainment bias, rapid hill climbing, and default parameters. After 4 iterations, Gubbins reached a stable tree topology and determined regions of genetic recombination. We visualized the resulting phylogenetic tree, isolate metadata, core genome SNPs, and recombination sites by using Phandango version 1.3.0 (*23*).

### Detection of Antimicrobial Resistance Genes

To detect the presence of antimicrobial resistance genes, we used BLASTN to search the assembled contigs of 27 *S. pneumoniae* against the reference sequences reported in previous studies (*3*,*24**–**26*). Our search included *ermB*, *cat*, *mef*(*A/E*), *tetO*, *tetM*, *folA*, *folP*, *rpoB*, *gyrA*, *gyrB*, *parC*, *parE*, and other Tn916-like transposon typing genes, including *aphA3*, *int*-Tn916, *xis*-Tn916, *tnpR*-Tn916, and *tnpA*-Tn916. We validated antimicrobial resistance genes and Tn916-like transposon types by using PCR (Appendix 1 Table 1).

### Statistical Analysis

We compared categorical variables by using χ^2^ test or 2-tailed Fisher exact test, when appropriate. We considered p<0.05 statistically significant. We performed analyses by using SPSS Statistics 15.0 (IBM, https://www.ibm.com).

## Results

### Isolate Demographics and Penicillin and Meropenem Susceptibility

We recovered 206 nonrepetitive *S. pneumoniae* isolates collected during the study: 136 from Linkou CGMH and 70 from Kaohsiung CGMH. Isolates were collected from blood (n = 178, 86.4%), cerebrospinal fluid (n = 6, 2.9%), and pleural fluid (n = 10, 4.9%). We grouped isolates from patients in the following age groups: <5 years of age, 22.8%; 5–64 years of age, 49%; and ≥65 years of age, 28.2%. Most (64.1%) isolates were from male patients. Serotype 19A (17.5%) was most common, followed by 15B/C (14.1%), 15A (9.7%), 14 (8.7%), and 19F (8.7%) (Table 1). Most (84.5%) isolates had an MIC of >0.06 mg/L for penicillin, and 5.3% had an MIC of >2 mg/L for penicillin. The MIC_50_ (MIC for 50% of the strains) for penicillin was 1 mg/L and the MIC_90_ (MIC for 90% of the strains) was 2 mg/L. Serotypes 19A (n = 36), 15B/C (n = 27), and 15A (n = 18) accounted for 46.6% of the isolates with an MIC >0.06 mg/L for penicillin. Isolates with an MIC >2 mg/L for penicillin were from serotypes 19A (n = 5), 19F (n = 4), 15B (n = 1), and 15A (n = 1). 

**Table 1:** Serotype distribution and penicillin and meropenem susceptibility rates of invasive *Streptococcus pneumoniae* isolates from 2 tertiary hospitals in Taiwan, 2013−2017*†

Serotype	Isolates, no.	No. (%) isolates										
		Meropenem				Penicillin, meningitis				Penicillin, nonmeningitis		
		S	I	R		S	I	R		S	I	R
PCV7												
4	4	3 (75)	1 (25)	0		3 (75)	NC	1 (25)		4 (100)	0	0
6B	8	6 (75)	2 (25)	0		1 (12.5)	NC	7 (87.5)		8 (100)	0	0
9V	2	2 (100)	0 (0)	0		2 (100)	NC	0		2 (100)	0	0
14	18	4 (22.2)	14 (77.8)	0		1 (5.6)	NC	17 (94.4)		18 (100)	0	0
18C	0	0	0	0		0	NC	0		0	0	0
19F	18	3 (16.7)	8 (44.4)	7 (38.9)		1 (5.6)	NC	17 (94.4)		14 (77.8)	4 (22.2)	0
23F	10	5 (50)	5 (50)	0		0	NC	10 (100)		10 (100)	0	0
Subtotal	60	23 (38.3)	30 (50)	7 (11.7)		8 (13.3)	NC	52 (86.7)		56 (93.3)	4 (6.7)	0
PCV13−non-PCV7												
1	1	0	1 (100)	0		0	NC	1 (100)		1 (100)	0	0
3	15	15 (100)	0	0		9 (60)	NC	6 (40)		15 (100)	0	0
5	0	0	0	0		0	NC	0		0	0	0
6A	7	3 (42.9)	4 (57.1)	0		1 (14.3)	NC	6 (85.7)		7 (100)	0	0
7F	0	0	0	0		0	NC	0		0	0	0
19A	36	4 (11.1)	13 (36.1)	19 (52.8)		0	NC	36 (100)		31 (86.1)	5 (13.9)	0
Subtotal	59	22 (37.3)	18 (30.5)	19 (32.2)		10 (16.9)	NC	49 (83.1)		54 (91.5)	5 (8.5)	0
PPV23−non-PCV13												
8	0	0	0	0		0	NC	0		0	0	0
10A	0	0	0	0		0	NC	0		0	0	0
11A	4	2 (50)	1 (25)	1 (25)		2 (50)	NC	2 (50)		4 (100)	0	0
15B	17	1 (5.9)	8 (47.1)	8 (47.1)		0	NC	17 (100)		16 (94.1)	1 (5.9)	0
20	0	0	0	0		0	NC	0		0	0	0
22F	3	3 (100)	0	0		3 (100)	NC	0		3 (100)	0	0
33F	0	0	0	0		0	NC	0		0	0	0
Subtotal	24	6 (25)	9 (37.5)	9 (34.6)		5 (19.2)	NC	19 (79.2)		23 (95.8)	1 (3.9)	0
NVT												
6C	1	1 (100)	0	0		0	NC	1 (100)		1 (100)	0	0
7B/C	2	2 (100)	0	0		1 (50)	NC	1 (50)		2 (100)	0	0
9A/L	2	2 (100)	0	0		1 (50)	NC	1 (50)		2 (100)	0	0
11B	1	1 (100)	0	0		1 (100)	NC	0		1 (100)	0	0
11C	1	1 (100)	0	0		1 (100)	NC	0		1 (100)	0	0
12	2	0	2 (100)	0		0	NC	2 (100)		2 (100)	0	0
15A	20	7 (35)	12 (60)	1 (5)		2 (10)	NC	18 (90)		19 (95)	1 (5)	0
15C	12	2 (16.7)	8 (66.7)	2 (16.7)		2 (16.7)	NC	10 (83.3)		10 (100)	0	0
15F	1	0	1(100)	0		0	NC	1 (100)		1 (100)	0	0
18F	1	0	1 (100)	0		0	NC	1 (100)		1 (100)	0	0
19B	1	1 (100)	0	0		0	NC	1 (100)		1 (100)	0	0
23A	14	10 (71.4)	3 (21.4)	1 (7.2)		0	NC	14 (100)		14 (100)	0	0
23B	2	1 (50)	1 (50)	0		0	NC	2 (100)		2 (100)	0	0
35A/C	2	1 (50)	1 (50)	0		1 (50)	NC	1 (50)		2 (100)	0	0
35B	1	1 (100)	0	0		0	NC	1 (100)		1 (100)	0	0
Subtotal	63	30 (47.6)	29 (46)	4 (6.3)		9 (14.3)	NC	54 (85.7)		58 (98.3)	1 (1.7)	0

*I, intermediate; NC, not calculated; NVT, nonvaccine type; PCV7, 7-valent pneumococcal conjugate vaccine; PCV13, 13-valent pneumococcal conjugate vaccine; PPV23, 23-valent pneumococcal polysaccharide vaccine; R, resistant; S, susceptible. †MICs for meropenem: S, <0.25; I, 0.5; R, >1; for penicillin, meningitis: S, <0.06; R, >0.12; and for penicillin, nonmeningitis: S, <2; I, 4; R, >8.

**Figure 1:**
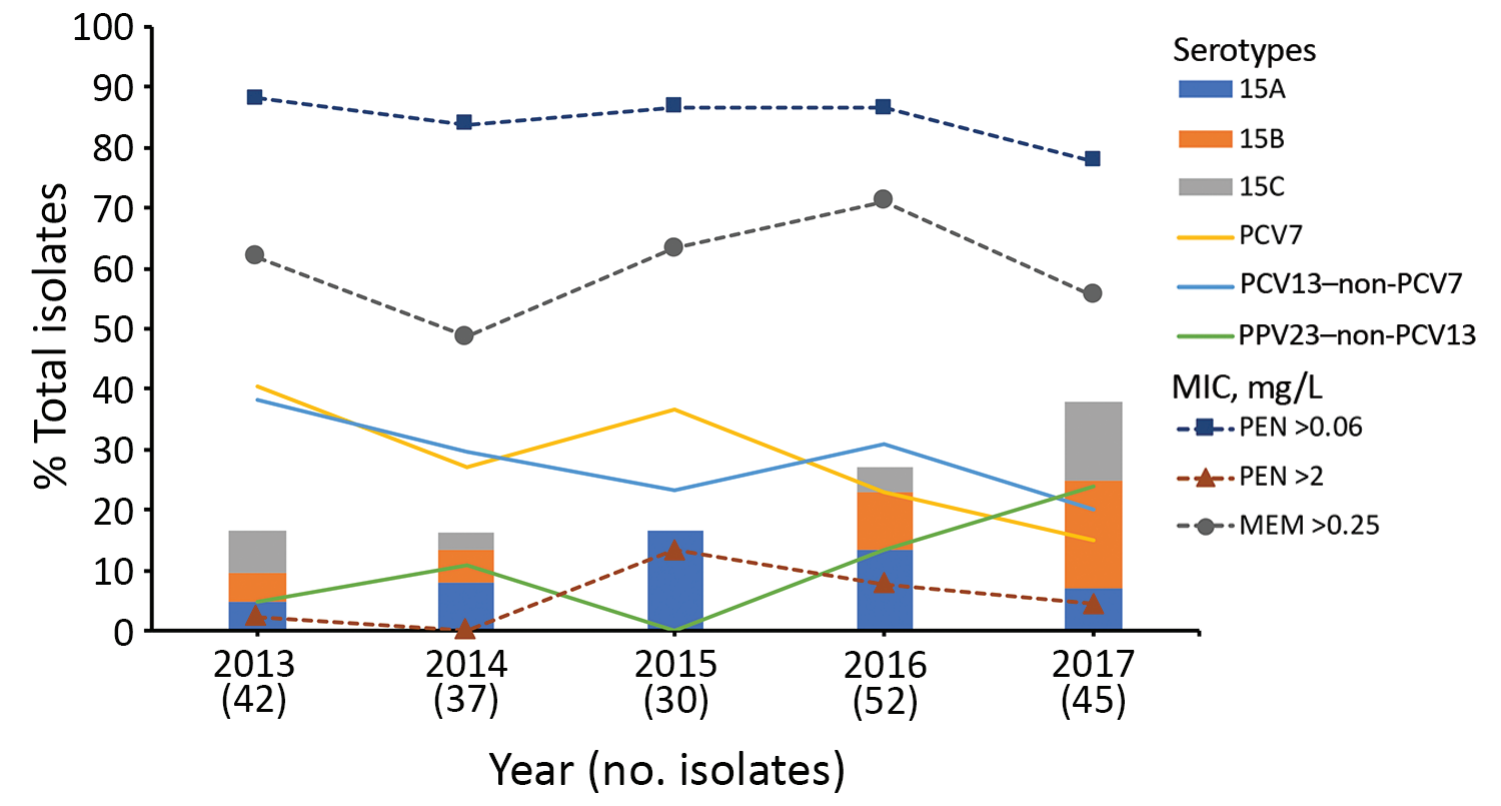
Serotype changes in *Streptococcus pneumoniae* strains isolated in Taiwan, 2013–2017. The prevalence of strains with an MIC >0.06 mg/L for penicillin was sustained during the study. PCV7 and PCV13–non-PCV7 serotypes decreased (p = 0.009) over time. In contrast, PPV23 serotypes and the non-PCV13 serotypes 15B/C increased (p = 0.002) and the rate of serotype 15A fluctuated. PCV7, 7-valent pneumococcal conjugate vaccine; PCV13, 13-valent pneumococcal conjugate vaccine; PPV23, 23-valent pneumococcal polysaccharide vaccine; PEN, penicillin; MEM, meropenem.

The rate of nonsusceptibility to meropenem was 60.7%; the MIC_50_ for meropenem was 0.5 mg/L and the MIC_90_ was 1 mg/L. PCV13 serotypes had high rates of meropenem nonsusceptibility, including 88.9% of 19A, 83.3% of 19F, and 77.8% of 14, as did non-PCV13 serotypes, including 94.1% of 15B, 83.3% of 15C, and 65% of 15A (Table 1). The prevalence of the 7-valent PCV (PCV7) and PCV13 serotypes decreased, but 23-valent pneumococcal polysaccharide vaccine (PPV23) serotypes increased from 4.8% of isolates in 2013 to 24.4% in 2017 (p = 0.009; Figure 1). The prevalence of non-PCV13 serotypes 15A and 15B/C increased from 16.7% of isolates in 2013 to 37.8% in 2017 (p = 0.08; Figure 1).

### MLST and PBP Allelic Profiles of Meropenem-Nonsusceptible Pneumococcal Isolates

The mechanisms of penicillin resistance in *S. pneumoniae* have been well studied, but data have been published on the mechanisms of meropenem resistance. We aimed to evaluate the role of PBP in meropenem resistance mechanisms. 

We used MLST to evaluate the clonal relatedness of 125 meropenem-nonsusceptible isolates. Most (n = 94) isolates were from 3 major clonal complexes (CCs): CC320, including ST320, ST236, ST271, ST1464, ST7122, ST12444, and ST14357; CC81, including ST81, ST83, and ST14359; and CC63, including ST63 and ST14313 (Appendix 1 Table 3). CC320 was associated with serogroup 19, but CC81 and CC63 were associated with more diverse serotypes. We noted that the prevalence of IPD caused by serotypes 15B/C-ST83 increased from 7.1% (3/42) of isolates in 2013 to 26.7% (12/45) in 2017 (p = 0.004). Clonal expansion of 15B/C-ST83 appeared to be associated with an increase in meropenem nonsusceptibility and in the number of cases of IPD caused by serotypes 15B/C.

Among meropenem-nonsusceptible isolates, we noted 3 PBP1a, 2b, 2x allelic profiles, 13:11:16, 15:12:18, and 13:new1:43. Isolates of 19A-ST320 harbored most 13:11:16 allele profiles (n = 27); 15B/C-ST83 harbored more 15:12:18 profiles (n = 23); and 15A-ST63 harbored more 13:new1:43 profiles (n = 10) (Appendix 1 Table 3). 

We identified several new PBP1a, 2b, and 2x types in this study (Appendix 1 Tables 4–6) and we examined the relatedness of the amino acid (aa) sequences of PBP1a, 2b, and 2x among meropenem-nonsusceptible isolates (Appendix 1 Figure 1). The PBP2x sequences of all meropenem-nonsusceptible isolates had T338A, R384G, and D567N aa substitutions; all PBP2b sequences had the Q432A/L and T451A aa substitutions; and all PBP1a sequences had E388D, E397I/V, N405S/D, G414A/V, N443D, and D473N/S aa substitutions (Appendix 2, http://wwwnc.cdc.gov/EID/article/26/4/19-0717-App2.xlsx).

### Effect of PBP Variants on the MIC of Meropenem for *S. pneumoniae* R6

The meropenem-susceptible pneumococcal strain R6 was transformed with individual entire *pbp* genes, as well as different combinations of the *pbp1a*, *pbp2b*, and *pbp2x* genes from a 15A-ST63 clinical isolate with a PBP profile of 13:new1:43 that had the highest MIC for penicillin and meropenem in our study (Table 2). No isolates of strain R6 that had *pbp2x* or *pbp1a* transformants had an increased MIC for meropenem, but when strain R6 was transformed with *pbp2b*, R6-2B transformants had an increased MIC of 0.06 mg/L for meropenem. DNA sequence analysis of the *pbp2b* gene from the R6-2B transformants confirmed that all the aa substitutions occurred in the PBP2b transpeptidase domain. Transformation of the R6-2B transformants with *pbp2x* generated R6-2B-2X transformants with an increased MIC of 0.25 mg/L for meropenem. DNA analysis of the *pbp2x* gene from the R6-2B-2X transformants revealed they possessed an additional 21 aa substitutions outside the transpeptidase domain of PBP2x. Transformation of the R6–2B-2X transformants with *pbp1a* produced R6-2B-2X-1A transformants and that had an increased MIC of 0.5 mg/L for meropenem. DNA sequence analysis of the *pbp1a* gene from the R6-2B-2X-1A transformants confirmed that the entire type 13 *pbp1a* was transformed. PBP2b and PBP2x appear to be involved in increased meropenem resistance, and PBP1a appears to be associated with breakthrough meropenem susceptibility.

**Table 2:** Pneumococcal transformation study results for study of invasive *Streptococcus pneumoniae* isolates from 2 tertiary hospitals in Taiwan, 2013–2017*

Recipient	Transforming DNA	Transformant name	Colony formation	MIC of transformants, mg/L		Integration of altered PBP in transformants	PBP profile 1A:2B:2X
				Meropenem	Penicillin		
R6	NA	R6-WT	ND	0.015	0.015	ND	2:0:2
15A-ST63	NA	15A-WT	ND	1	4	ND	13:new1:43
R6	*pbp1a*	R6–1A	−	ND	ND	ND	ND
R6	*pbp2b*	R6–2B	+	0.06	0.015	Yes, PBP2b	2:new1:2
R6	*pbp2x*	R6–2X	−	ND	ND	ND	ND
R6–2B transformants	*pbp2x*	R6–2B-2X	+	0.25	0.125	Yes, PBP2x	2:new1:43
R6–2B transformants	*pbp1a*	R6–2B-1A	−	ND	ND	ND	ND
R6–2B-2X transformants	*pbp1a*	R6–2B-2X-1A	+	0.5	1	Yes, PBP1a	13:new1:43

*NA, not applicable; ND, not done; PBP, penicillin binding protein; +, positive for colony formation on antimicrobial-containing agar plate; −, negative for colony formation on antimicrobial-containing agar plate.

### Antimicrobial Resistance Genes and Phylogenomic Tree Analysis

All 15B/C-ST83 isolates in this study had *tetM*, *ermB*, *cat*, and *folA* mutations and *folP* insertions, but none had *ermTR*, *mef*(*A/E*), or *tetO* genes; none carried mutations in *rpoB*, *gyrA*, *gyrB*, or *parE*, but 2 had a mutation in the *parC* gene. Tn6002, carrying the *ermB-*mediated macrolide resistance gene, was detected in all 15B/C-ST83 isolates in our study (Table 3; Appendix 1 Table 7). 

**Table 3:** Antimicrobial resistance genes and Tn916-like transposon gene in 24 meropenem-nonsusceptible *Streptococcus pneumoniae* 15B/C-ST83 clinical isolates*

Isolate name, serotype			PBP profile 1a:2b:2x	Antimicrobial resistance genes														
				*tetM*	*tetO*	*ermB*	*ermTR*	*mef* *(A/E)*	Tn of Tn916-like	*cat*	CFT	Mutations						
	MIC, mg/L											*folA*†	*folP*‡	*gyrA*	*gyrB*	*parC*§	*parE*	*rpoB*
	PEN	MEM																
2013																		
B1136, 15C	1	0.5	15:12:18	+	−	+	−	−	Tn6002	+	R	+	+	−	−	−	−	−
B999-49, 15B	1	0.5	15:12:18	+	−	+	−	−	Tn6002	+	R	+	+	−	−	−	−	−
B1300936, 15C	2	0.5	15:12:18	+	−	+	−	−	Tn6002	+	R	+	+	−	−	−	−	−
2014																		
1018-71,15B	2	0.5	15:12:18	+	−	+	−	−	Tn6002	+	R	+	+	−	−	−	−	−
1025-08, 15B	2	0.5	15:12:18	+	−	+	−	−	Tn6002	+	R	+	+	−	−	−	−	−
1054-32, 15C	2	0.5	15:12:18	+	−	+	−	−	Tn6002	+	R	+	+	−	−	−	−	−
2016																		
B1675, 15B	2	1	15:12:18	+	−	+	−	−	Tn6002	+	R	+	+	−	−	−	−	−
B0236, 15B	2	1	15:12:18	+	−	+	−	−	Tn6002	+	R	+	+	−	−	−	−	−
1148-64, 15C	2	0.5	15:12:18	+	−	+	−	−	Tn6002	+	R	+	+	−	−	−	−	−
1217-52, 15B	1	0.5	15:12:18	+	−	+	−	−	Tn6002	+	R	+	+	−	−	−	−	−
K54, 15B	2	1	15:12:18	+	−	+	−	−	Tn6002	+	R	+	+	−	−	−	−	−
K55, 15B	2	1	15:12:18	+	−	+	−	−	Tn6002	+	R	+	+	−	−	−	−	−
2017																		
B2581, 15B	2	1	15:12:18	+	−	+	−	−	Tn6002	+	R	+	+	−	−	+	−	−
B5939, 15B	2	0.5	15:12:18	+	−	+	−	−	Tn6002	+	R	+	+	−	−	+	−	−
B4505, 15C	2	0.5	15:12:18	+	−	+	−	−	Tn6002	+	R	+	+	−	−	−	−	−
B1757, 15C	2	1	15:12:18	+	−	+	−	−	Tn6002	+	R	+	+	−	−	−	−	−
B3127, 15C	2	0.5	15:12:18	+	−	+	−	−	Tn6002	+	R	+	+	−	−	−	−	−
B2404, 15B	2	0.5	15:12:18	+	−	+	−	−	Tn6002	+	R	+	+	−	−	−	−	−
B8812, 15B	2	0.5	15:12:18	+	−	+	−	−	Tn6002	+	R	+	+	−	−	−	−	−
1244-61, 15B	2	0.5	15:12:18	+	−	+	−	−	Tn6002	+	R	+	+	−	−	−	−	−
1267-33, 15B	2	1	15:12:18	+	−	+	−	−	Tn6002	+	R	+	+	−	−	−	−	−
1276-72, 15C	2	1	15:12:18	+	−	+	−	−	Tn6002	+	R	+	+	−	−	−	−	−
K72, 15B	2	1	15:12:18	+	−	+	−	−	Tn6002	+	R	+	+	−	−	−	−	−
K110, 15B	4	1	15:12:18	+	−	+	−	−	Tn6002	+	R	+	+	−	−	−	−	−

*CFT, ceftriaxone; MEM, meropenem; PEN, penicillin; R, resistant; +, positive; −, negative. †I100L substitution. ‡Insertion of 1 codon between bases 168 and 201. §S79Y substitution.

**Figure 2:**
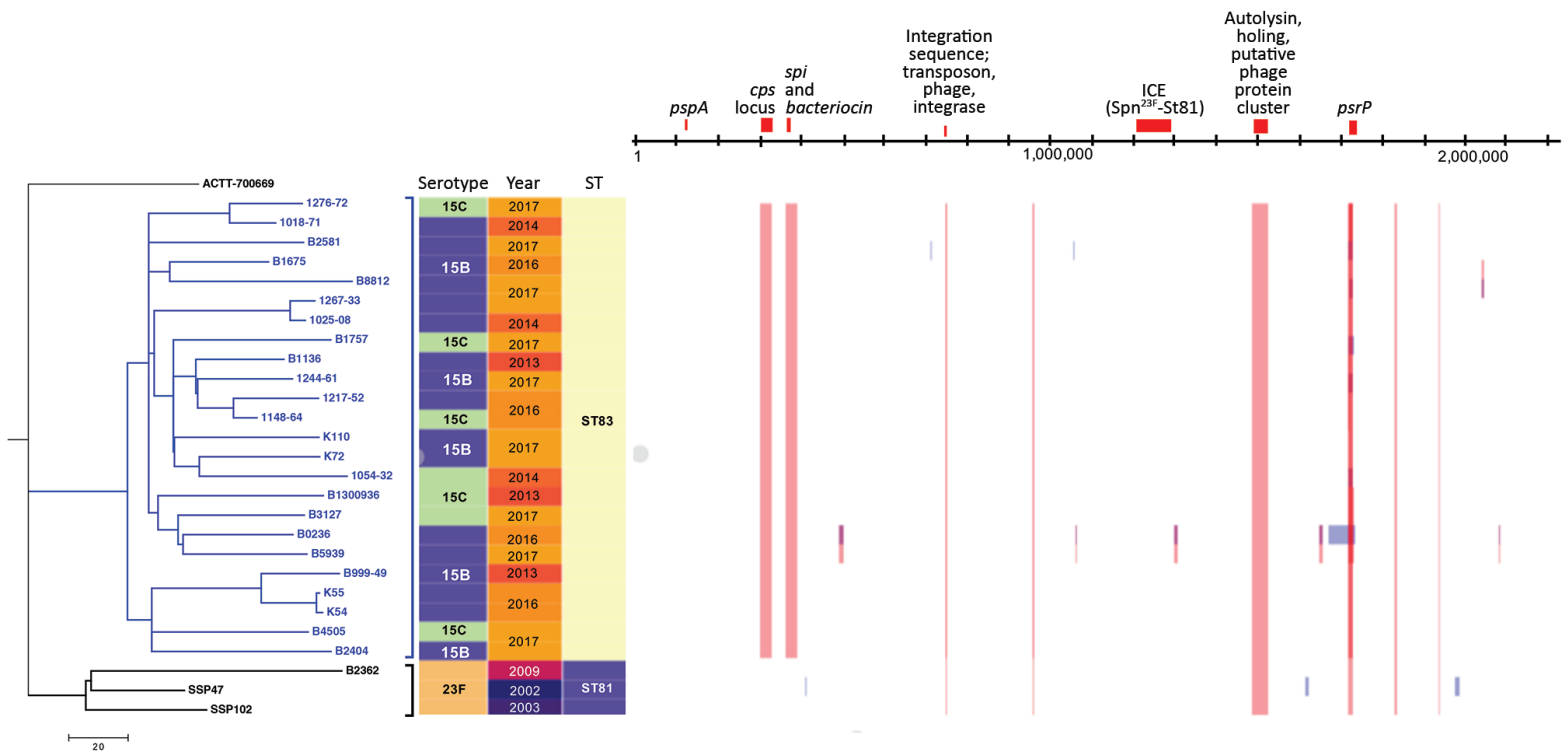
Phylogenic tree and recombination sites of 24 *Streptococcus pneumoniae* serotype 15B/C-ST83 isolates collected from Taiwan during 2013–2017 and 3 serotype 23F-ST81 isolates collected during an earlier study in Taiwan. Phylogenic tree compares these 27 isolates against the reference strain, *S. pneumoniae* Spain^23F^-ST81 ATCC-700669 (GenBank accession no. FM211187). Blue text represents the 15B/C-ST83 serotype isolates from this study, which cluster together in the tree. Recombination positions are based on the 2,221,315-bp of the reference strain. Scale bar indicates nucleotide substitutions per site. ST, sequence type.

Using ATCC-700669 as an outgroup, we constructed a whole-genome phylogenic tree for the 24 15B/C-ST83 isolates and 3 23F-ST81 isolates (*10*). All 15B/C-ST83 isolates clustered in a clade, and the 23F-ST81 isolates clustered in another clade (Figure 2). We determined 12 recombination sites between all 15B/C-ST83 isolates, and we observed 7 of them in the 3 23F-ST81. We noted 5 specific recombination sites in all 15B/C-ST83 isolates: pneumococcal surface protein A (*pspA*) at positions 124732–124902; the capsule polysaccharide (*cps*) locus at positions 302151–329719; *spi* and *bacteriocin* at positions 362492–389805; putative DNA binding protein at positions 2094386–2095149; and choline binding protein A (*cbpA*) at positions 2171700–2171829 (Appendix 1 Table 8). We also constructed a recombination-free tree in Gubbins that demonstrates the same phylogenetic groups (Appendix 1 Figure 2).

## Discussion

Widespread administration of PCVs decreases the incidence of antimicrobial drug–resistant *S. pneumoniae* because it targets serotypes that carry multiple genetic determinants of antimicrobial drug resistance that usually cause human disease (*27*). We found that previously successful clones changed their capsular types or served as a gene pool reservoir and donated genes associated with antimicrobial drug resistance, continuing their spread to other clones in the community. After universal vaccination with PCV13 in Taiwan, meropenem-nonsusceptibility was attributable to the emergence and spread of non-PCV13 serotype 15B/C-ST83 and 15A-ST63 clones, in addition to the PCV13 serotype 19A-ST320. 

Of note, 15B/C-ST83 showed an increasing trend during 2013–2017. Rates of carriage and invasive disease caused by nonvaccine serotype 15A and 15B/C have increased with increased use of PCV13 (*28*). The serotype 15A-ST63 clone we detected in Taiwan was the same clone detected in Japan, which carries an identical whole PBP1a (type 13) and includes a new PBP2b associated with resistance to penicillin and meropenem. Alterations in PBP1a, PBP2b, and PBP2x are associated most often with β-lactam resistance (*29*). Limited data are available on the mechanism underlying meropenem resistance in pneumococcus. We demonstrated that PBP2b has a high affinity for meropenem and sets the susceptibility threshold of R6. Hence, transfer of the *pbp2b* gene to R6 decreased its susceptibility to meropenem, which resulted in an incremental increase in resistance after sequential introduction of the *pbp2x* and *pbp1a*. Acquisition of the PBP2b and PBP2x variants appears to be a prerequisite for creating the PBP1a variants that confer high-level resistance to meropenem, similar to the stepwise development of penicillin resistance (*30*). However, the MIC levels of resistance of the last R6-2B-2X-1A transformant for penicillin and meropenem were not as high as those of its parent strain, 15A-ST63. The results of this transformation experiment suggested that other PBP and non-PBP contributors are involved in the resistance mechanism.

In Hong Kong, ST8589 and ST199 were major clones in serotype 15B/C, and both were susceptible to β-lactam (*28*). In the United States, ST199 susceptible to β-lactam and ST3280 with low-level β-lactam resistance (MIC 0.12–1 mg/L) were major genotypes of serotype 15B/C (*11*). In Japan, most 15B/C isolates were ST199 with low-level β-lactam resistance (*31*). ST199 primarily circulated as either serotype 19A or 15B/C. After the use of PCV7 in the United States, ST199 with low-level β-lactam resistance was the most prevalent clone among 19A isolates before 2005, after which it was outcompeted by highly resistant ST320, presumably due to pressure from antimicrobial drugs (*32*). 

In Taiwan, we did not find ST199 among any isolates (*12*,*33*), and serotype 15B/C-ST83 was the predominant clone. Serotype 15B/C-ST83 rarely was isolated in the United States, Japan, and Hong Kong (only 1–3 isolates among >100) (*11*,*28*,*31*), but is characterized exclusively with high-level β-lactam resistance (MIC ≥2 mg/L) and *ermB-* or *mef*(*A/E*)-mediated macrolide resistance. ST83 is a single-locus variant of ST81 grouped into the PMEN1 lineage. PMEN1, predominantly circulating as the PCV7 vaccine serotype 23F, was one of the pandemic penicillin-resistant clones identified in Spain in the 1980s that subsequently spread worldwide (*34*). ST81 shows high rates of carriage and disease and possesses the ICE, MM1 phage, Na^+^-dependent ATPase island, and TprA2/PhrA2 genomic regions, which are associated with increased colonization and virulence and led to their ecologic success and dominance (*35*,*36*). The Spain^23F^-ST81 clone was resistant not only to tetracycline and chloramphenicol but also frequently developed resistance to fluoroquinolone, rifampin, and macrolides (*37*,*38*). In addition, members of the PMEN1 lineage frequently switched to alternative capsular types and rapid genomic evolution through recombination that occurred in response to the selective pressure exerted by vaccines and antimicrobial drugs (*39*). 

We found a trend of 15B/C-ST83 clonal expansion among cases of IPD in Taiwan during 2013–2017. We also noted an increased prevalence of 15C-ST83. Reversible switching between serotypes 15B and 15C occurs during natural infection (*40*) and it is biologically plausible that the prevalence of 15C increased after 15B surged. 15B and 15C serotypes are distinguished by the presence or absence of an *O*-acetyl group attached to the capsular polysaccharide, which is ascribed to variation in the short tandem TA repeats in the *O*-acetyltransferase gene (*41*). We used WGS to confirm that the 15C isolates in our study had 7–13 tandem TA repeats and 15B isolates had 8 tandem TA repeats in the *O*-acetyltransferase gene. Of note, the functional antibodies generated against serotype 15B after administration of PPV23 have low cross-reactivity with serotype 15C (*42*). 

Aside from the longest recombination fragment *cps* locus in 15B/C-ST83, we detected other recombination sites by WGS, including *pspA* and *cbpA*, which are reported to be recombination sites in the ST81 lineage (*39*,*43*), and the *spi* allele, which shifted from ST81 to ST83 and is the allele that most distinguishes the difference between ST83 and ST81. In addition, we detected the antibacterial toxin *bacteriocin*, which enables 1 strain to predominate over others and mediates intraspecies competition during nasopharyngeal colonization in human hosts (*44*). In addition, all 15B/C-ST83 isolates in our study were resistant to macrolide and harbored the Tn6002 element, via *ermB*, *tetM*, *int*-Tn916, and *xis*-Tn916. Tn6002 has been identified as the most common Tn916-like element among *ermB*-carrying strains (*45*).

Serotypes differ greatly in their carriage prevalence, mainly because of the biochemical structure of a specific capsular polysaccharide (*46*). Nonvaccine types with polysaccharides that have fewer carbons and low energy expenditure per repeat unit, such as 15A, 15B/C, and 35B, have become the next generation of colonizers after the vaccine types diminished (*46*). In this study, under the selective pressure exerted by vaccines and antimicrobial drugs, the 15A-ST63 clone from Taiwan also acquired PBP1a from 19A-ST320, similar to the 15A-ST63 clone from Japan (*4*). The meropenem-nonsusceptible 15A-ST63 clone identified in this study and a study from Japan might have been derived from the same ancestor or could have generated simultaneously by parallel evolution (*4*). The capsular switching recombination event in the ancestor, Spain^23F^-ST81 clone, to 15B/C-ST83 continue the spread of PMEN1 lineage. Our data regarding the epidemiology of IPD aligns with carriage data (*47*) that showed that PCV reduced the overall carriage and disease, but antimicrobial resistance in pneumococcus was still high. 

Although our study was performed at just 2 medical centers, the prevalent serotypes after implementation of PCV13 and the rates of β-lactam resistance we observed are consistent with national data for Taiwan (*6*). The PMEN1 lineage was not eliminated by PCV, and its spread is concerning. Rational antimicrobial drug use and continued surveillance are necessary to monitor the epidemiologic trends and protect public health.

Appendix 1 Additional information on the spread of meropenem-resistant *Streptococcus pneumoniae* Spain23F-ST81 in Taiwan.

Appendix 2 Amino acid substitutions in penicillin-binding proteins of meropenem-resistant *Streptococcus pneumoniae* Spain23F-ST81 in Taiwan.
