# Supplementary material for: Genomic Insight into the Spread of Meropenem-Resistant Streptococcus pneumoniae Spain23F-ST81, Taiwan
Source: Emerg Infect Dis. 2020 Apr;26(4):711–20. doi: 10.3201/eid2604.190717 (PMC7101100; doi:10.3201/eid2604.190717)
Supplement: Appendix 1 — Additional information on the spread of meropenem-resistant Streptococcus pneumoniae Spain23F-ST81 in Taiwan. [file 19-0717-Techapp-s1.pdf]

Article DOI: https://doi.org/10.3201/eid2604.190717

Genomic Insight into the Spread of Meropenem-Resistant *Streptococcus pneumoniae* Spain^{23F}-ST81, Taiwan

Appendix 1

Appendix 1 Table 1. Primers used in this study

Primer name	Primer sequence (5′→3′)	Purpose	Reference no.
pbp1a-F	CCAGCAACAGGTGAGAGTC	PCR	(*1*)
pbp1a-R	GTAAACACAAGCCAAGACAC		
pbp2b-F	CCGTCTTAATCCCGATACC	PCR	(*1*)
pbp2b-R	ATTTTTGGGTGACTTGTTGAG		
pbp2x-F	GGAATTGGTGTCCCGTAAGC	PCR	(*1*)
pbp2x-R	CATCTGCTGGCCTGTAATTTG		
pbp1a-F2	CCWGTCCATACAGCCATWG	Sequencing	This study
pbp1a-R2	CTTCTCAGCAAGAAACCT		
pbp2b-F2	TATACTCTTTKGGWAYAA	Sequencing	This study
pbp2b-R2	CTGACTTATGTGAGCATC		
pbp2x-F2	ACGTGGGACTATTTATGAC	Sequencing	This study
pbp2x-R2	TTGGTAGAACCAACCAAG		
ermB-F	TGGTATTCCAAATGCGTAATG	PCR	(*2*)
ermB-R	CTGTGGTATGGCGGGTAAGT		
mef(*A/E*)-F	CAATATGGGCAGGGCAAG	PCR	(*2*)
mef(*A/E*)-R	AAGCTGTTCCAATGCTACGG		
cat-F	TTAGGYTATTGGGATAAGTTA	PCR	(*3*)
cat-R	CATGRTAACCATCACAWAC		
tetM-F	GTGGACAAAGGTACAACGAG	PCR	(*2*)
tetM-R	CGGTAAAGTTCGTCACACAC		
aphA3-F	TAAAAGATACGGAAGGAATGTCTC	PCR	(*2*)
aphA3-R	TCGACCGGACGCAGAAGGCAATGT		
int-Tn916-F	GCGTGATTGTATCTCACT	PCR	(*2*)
int-Tn916-R	GACGCTCCTGTTGCTTCT		
xis-Tn916-F	AAGCAGACTGAGATTCCTA	PCR	(*2*)
xis-Tn916-R	GCGTCCAATGTATCTATAA		
tnpR-F	CCAAGGAGCTAAAGAGGTCCC	PCR	(*2*)
tnpR-R	GTCCCGAGTCCCATGGAAGC		
tnpA-F	GCTTCCATGGGACTCGGGAC	PCR	(*2*)
tnpA-R	GCTCCCAATTAATAGGAGA		
folA-F	TTGCCAGCAGAATTGCAGCA	PCR	(*4*)
folA-R	AAATAGGTATCTCCTTCCACC		
folP-F	GATGAATGCATCGTGTCCATC	PCR	(*5*)
folP-R	CCGTCCGGTAGTTAGCAATCC		

Appendix 1 Table 2. Sequence Read Archive accession numbers used in this study*

Isolate name	Serotype	ST	Year	Accession no.
B1136	15C	83	2013	SRR8867345
B999-49	15B	83	2013	SRR8867344
B1300936	15C	83	2013	SRR8867343
1018-71	15B	83	2014	SRR8867342
1025-08	15B	83	2014	SRR8867349
1054-32	15C	83	2014	SRR8867348
B1675	15B	83	2016	SRR8867347
B0236	15B	83	2016	SRR8867346
1148-64	15C	83	2016	SRR8867351
1217-52	15B	83	2016	SRR8867350
K54	15B	83	2016	SRR8867360
K55	15B	83	2016	SRR8867359
B2581	15B	83	2017	SRR8867362
B5939	15B	83	2017	SRR8867361
B4505	15C	83	2017	SRR8867364
B1757	15C	83	2017	SRR8867363
B3127	15C	83	2017	SRR8867366
B2404	15B	83	2017	SRR8867365
B8812	15B	83	2017	SRR8867368
1244-61	15B	83	2017	SRR8867367
1267-33	15B	83	2017	SRR8867355
1276-72	15C	83	2017	SRR8867356
K72	15B	83	2017	SRR8867357
K110	15B	83	2017	SRR8867358
SSP102	23F	81	2003	SRR8867352
SSP47	23F	81	2002	SRR8867353
B2362	23F	81	2009	SRR8867354

*ST, sequence type. Sequence Read Archive, http://www.ncbi.nlm.nih.gov/sra.

Appendix 1 Table 3. Multilocus sequence type (MLST) analysis and penicillin binding-protein (PBP) allelic profile of 125 meropenem-nonsusceptible pneumococcal isolates

			MIC (mg/L)						PBP profile			
			Meropenem		Penicillin							
Serotype	ST no.	No. of isolates	0.5	≥1	≤0.06	≤2	4	≥8	PBP1a	PBP2b	PBP2x	no.
14	63	1	1	0	0	1	0	0	13	new1	43	1
	83	2	2	0	0	2	0	0	15	12	18	1
									60	16	143-like	1
	876	3	3	0	0	3	0	0	15	12	18	1
									60	16	143-like	2
	2652	3	3	0	0	3	0	0	15	31	7	1
									15	31	18	2
	3652	1	1	0	0	1	0	0	15	31	18	1
	14312	1	1	0	0	1	0	0	60	16	143-like	1
	14313	1	1	0	0	1	0	0	24	53	77	1
	14314	1	1	0	0	1	0	0	60	16	143-like	1
	14315	1	1	0	0	1	0	0	15	31	18	1
15A	63	13	12	1	0	11	2	0	15	12	18	1
									13	new1	43	10
									13	new1	16	1
									13	new1-like	43-like	1
15B	83	16	8	8	0	15	1	0	15	12	18	16
15C	83	8	6	2	0	8	0	0	15	12	18	7
									15	12	36	1
	4743	1	1	0	0	1	0	0	15	31	18	1
	14359	1	0	1	0	1	0	0	15	12	18	1
19A	320	30	12	18	0	25	5	0	13	11	16	27
									13	11	16-like	2
									13	11	16-like-2	1
	876	1	1	0	0	1	0	0	60	16	143-like	1
	7122	1	0	1	0	1	0	0	13	11	16	1
19F	236	3	2	1	0	3	0	0	13	new1	47	1
									13	16	8	1
									60	16	20	1
	271	2	1	1	0	2	0	0	13	11	33	1
									13	16	47	1
	320	3	3	0	0	3	0	0	13	11	33	1
									13	11	16	1
									13	49-like	16	1
	1464	2	1	1	0	1	1	0	13	49-like	154-like	2
	12444	3	0	3	0	0	3	0	13	37	112-like	3
	14358	1	1	0	0	1	0	0	13	16	100-like	1
	14357	1	0	1	0	1	0	0	13	11-like	112-like-2	1
23A	166	3	2	1	0	3	0	0	15	11	36-like	3
	242	1	1	0	0	1	0	0	13	31	73	1
23F	81	1	1	0	0	1	0	0	15	12	18	1
	166	1	1	0	0	1	0	0	15	11	36-like	1
	242	2	2	0	0	2	0	0	13	31	73	1
									13	31	20	1
	1437	1	1	0	0	1	0	0	7	74-like	new2	1
6A	76	1	1	0	0	1	0	0	25-like	46	36	1
	81	3	3	0	0	3	0	0	15	12	18	3
6B	720	1	1	0	0	1	0	0	13-like	31-like	73-like	1
	8526	1	1	0	0	1	0	0	34	11	8-like	1
Others												
1	63	1	1	0	0	1	0	0	13	new1	43	1
15F	63	1	1	0	0	1	0	0	13	new1	43	1
18F	81	1	0	1	0	1	0	0	15	12	18	1
11A	166	2	1	1	0	2	0	0	15	12	18	1
									15	12	8	1
23B	242	1	1	0	0	1	0	0	13	31	73	1
4	558	1	1	0	0	1	0	0	4	7	7	1
35A/C	9395	1	1	0	0	1	0	0	4	7	7	1
12	9395	1	1	0	0	1	0	0	4	7	7	1
	12446	1	1	0	0	1	0	0	4	7	7	1
Total		125	84	41	0	113	12	0				

Appendix 1 Table 4. Novel PBP1a transpeptidase domain sequences identified in this study

Sequences
>13-like<br>SMKPITDYAPALEYGVYDSTASIVHDVPYNYPGTDTPLYNWDHVYFGNITIQYALQQSRNVTAVETLNKVGLDRAKTFLNGLGIDYP<br>SMHYANAISSNTTESNKKYGASSEKMAAAYAAFANGGIYHKPMYINKIVFSDGSEKEFSDAGTRAMKETTAYMMTEMMKTVLTHGT<br>GRGAYLPWLPQAGKTGTSNYTDEEIEKYIKNTGYVAPDEMFVGYTRKYSMAVWTGYSNRLTPIVGDGFLVAAKVYRSMMTYLSEG<br>SNPEDWNIPEGLYRNGEFV
>25-like<br>AMKPITDYAPAIEYGVYDSTATMVNDIPYNYPGTSTPVYNWDRAYFGNITLQYALQQSRNVTAVETLNKVGLDRAKTFLNGLGIDYP<br>SMHYANAISSNTTESNKQYGASSEKMAAAYAAFANGGIYHKPMYINKVVFSDGSEKEFSDVGTRAMKETTAYMMTEMMKTVLAYG<br>TGRGAYLPWLAQAGKTGTSNYTDDEIEKHIKNTGYVAPDEMFVGYTRKYSMAVWTGYSNRLTPIVGDGFLVAAKVYRSMITYLSED<br>THPEDWNIPEGLYRNGEFV

Appendix 1 Table 5. Novel PBP2b transpeptidase domain sequences identified in this study

Sequences
>31-like<br>TNVFVPGSVVKAATISSGWENGVLSGNQTLTDQPIVFQGSAPIYSWYKLAYGSFPITAVEALEYSSNAYMVQTALGIMGQTYQPNM<br>FVGTSNLETAMGKLRATFGEYGLGAATGIDLPDESTGFVPKEYSFANYIANAFGQFDNYTPMQLAQYVATIANNGVRVAPRIVEGIY<br>GNNDKGGLGDLIQQLQPTEMNKVNISDSDMSILHQGFYQVAHGTSGLTTGRAFSNGALVSISGKTGTAESYVADGQQATNTNAVA<br>YAPSDNPQIAVAVVFPHNTN
>49-like<br>TNVFIPGSVVKAATISSGWENGVLSGNQTLTDQPIVFQGSAPIYSWYKLAYGSFPITAVEALEYSSNAYMVQTALGIMGQTYQPNMF<br>VGTSNLETAMGKLRATFGEYGLGAATGIDLPYESTGFVPKEYSFANYITNAFGQFDNYTPMQLAQYVATIANDGVRVAPRIVEGIYG<br>NNDKGGLGDLIQQLQPTEMNKVNISDSDMSILHQGFYQVSHGTSPLTTGRAFSDGATVSISGKTGTGESYVAGGQEANNTNAVAY<br>APTENPQIAVAVVFPHNTN
>new1-like<br>TNVFVPGSVVKAATISSGWENGVLSGNQTLTDQPIVFQGSAPIYSWYKLAYGSFPITAVEALEYSSNAYMVQTALGIMGQTYQPNM<br>FVGTSNLESAMEKLRSTFGEYGLGTATGIDLPDESTGFVPKEYSFANYITNAFGQFDNNTPMQLAQYVATIANNGVRVAPRIVEGIY<br>GNNDKGGLGDLIQQLQPTEMNKVNISDSDMSILHQGFYQVAHGTSGLTTGRAFSNGAAVSISGKTGTAESYVEGGQEANNTNAVA<br>YAPSDNPQIAVAVVFPHNTN

Appendix 1 Table 6. Novel PBP2x transpeptidase domain sequences identified in this study

Sequences
>8-like<br>GKDGIITYEKDRLGNIVPGTEQVSQQTVDGKDVYTTLSSPLQSFMETQMDAFLEKVKGKYMTATLVSAKTGEILATTQRPTFNADTK<br>EGITEDFVWRDILYQSNYEPGSAMKVMTLASSIDNNTFPSGEYFNSSEFKIADATTRDWDVNEGLTTGGMMTFLQGFAHSSNVGM<br>SLLEQKMGDATWLDYLKRFKFGVPTRFGLTDEYAGQLPADNIVSIAQSSFGQGISVTQTQMLRAFTAIANDGVMLEPKFISAIYDTNN<br>QSVRKSQKEIVGKPVSEEAASTTRNHMILVGTDPLYGTMYNHYTGKPIITVPGQNVAVKSGTAQIADEKNGGYLVGSTNYIFSAVTM<br>NPAENPDFILYV
>16-like<br>GTDGIITYEKDRVGNIVPGTELVSQQTVDGKDVYTTLSSPLQSFMETQMDAFLEKVKGKYMTATLVSAKTGEILATTQRPTFNADTK<br>EGITEDFVWRDILYQSNYEPGSAMKVMTLASSIDNNTFPSGEYFNSSEFKIADATTRDWDVNEGLTTGGMMTFLQGFAHSSNVGM<br>SLLEQKMGDATWLDYLKRFKFGVPTRFGLTDEYAGQLPADNIVSIAQSSFGQGISVTQTQMLRAFTAIANDGVMLEPKFISAIYDPNN<br>QSVRKSQKEIVGKPVSEDTASLTRTNMILVGTDPLYGTMYNHYTGKPIITVPGQNVAVKSGTAQIADEKNGGYLVGSTNYIFSAVTM<br>NPAENPDFILYV
>16-like-2<br>GTDGIITYEKDRVGNIVPGTELVSQQTVDGKDVYTTLSSPLQFFMETQMDAFLEKVKGKYMTATLVSAKTGEILATTQRPTFNADTK<br>EGITEDFVWRDILYQSNYEPGSAMKVMTLASSIDNNTFPSGEYFNSSEFKIADATTRDWDVNEGLTTGGMMTFLQGFAHSSNVGM<br>SLLEQKMGDATWLDYLKRFKFGVPTRFGLTDEYAGQLPADNIVSIAQSSFGQGISVTQTQMLRAFTAIANDGVMLEPKFISAIYDTNN<br>QSVRKSQKEIVGKPVSEDTASLTRTNMILVGTDPLYGTMYNHYTGKPIITVPGQNVAVKSGTAQIADEKNGGYLVGSTNYIFSAVTM<br>NPAENPDFILYV
>36-like<br>GTDGIITYEKDRVGNIVPGTELVSQQTVDGKDVYTTLSSPLQSFMETQMDAFLEKVKGKYMTATLVSAKTGEILATTQRPTFNADTK<br>EGITEDFVWRDILYQSNYEPGSAMKVMTLASSIDNNTFPSGEYFNSSEFKMADVTTRDWDVNGGLTTGGMMTFLQGFAHSSNVG<br>MSLLEQKMGDATWLDYLKRFKFGVPTRFGLTDEYAGQLPADNIVSIAQSSFGQGISVTQTQMLRAFTAIANDGVMLEPKFISAIYDT<br>NNQSVRKSQKEIVGNPVSKETASTTRNHMILVGTDPLYGTMYNHYTGKPIITVPGQNVAVKSGTAQIADEKNGGYLVGSTNYIFSAV<br>TMNPAENPDFILYV
>43-like<br>GTDGIITYEKDRLGNIVPGTEQVSQQTVDGKDVYTTISSTLQSFMETQMDAFLEKVKGKYMTATLVSAKTGEILATTQRPTFNADTKE<br>GITEDFVWRDILYQSNYEPGSAMKVMTLASSIDNNTFPSGEYFNSSEFKIADATTRDWDVNEGLTTGGMMTFLQGFAHSSNVGMS

Sequences
LLEQKMGDATWLDYLKRFKFGVPTRFGLTDEYAGQLPADNIVSIAQSSFGQGISVTQTQMLRAFTAIANDGVMLEPKFISAIYDTNN<br>QSVRKSQKEIVGNPVSKEAASTTRNHMLLVGTDPLYGTMYNHYTGKPIITVPGQNVAVKSGTAQIADEKNGGYLVGSTNYIFSAVTM<br>NPAENPDFILYV
>73-like<br>GTDGIITYEKDRLGNIVPGTEQVSQRTMDGKDVYTTISSPLQSFMETQMDAFLEKVKGKYMTATLVSAKTGEILATTQRPTFNADTK<br>EGITEDFVWRDILYQSNYEPGSAMKVMTLASSIDNNTFPSGEYFNSSEFKIADATIRDWDVNDGLTTGGMMTFLQGFAHSSNVGMS<br>LLEQKMGDATWLDYLKRFKFGVPTRFGLTDEYAGQLPADNIVSIAQSSFGQGISVTQTQMLRAFTAIANDGVMLEPKFISAIYDTNN<br>QSVRKSQKEIVGNPVSKEAASTTRNHMILVGTDPLYGTMYNHYTGKPIITVPGQNVAVKSGTAQIADEKNGGYLVGSTNYIFSVVTM<br>NPAENPDFILYV
>100-like<br>GTDGIITYEKDRVGNIVPGTELVSQQTVDGKDVYTTLSSPLQSFMETQMDAFLEKLKGKYMTATLVSAKTGEILATTQRPTFNADTK<br>EGITEDFVWRDILYQSNYEPGSGMKVMTLASSIDNNTFPSGEYFNSSEFKIADATTRDWDVNEGLTTGGMMTFLQSFAHSSNVGM<br>SLLEQKMGDATWLDYLKRFKFGVPTRFGLTDEYAGQLPADNIVSISQSSFGQGISVTQTQMLRAFTAIANDGVMLEPKFISAIYDTNN<br>QSVRKSQKEIVGNPVSKEAASTTRNHMILVGTDPLYGTMYNHYTGKPIITVPGQNVAVKSGTAQIADEKNGGYLVGSTNYIFSVVTM<br>NPAENPDFILYV
>112-like<br>GKDGIITYEKDRLGNIVPGTEQVSQQTVDGKDVYTTLSSPLQSFMETQMDAFLEKLKGKYMTATLVSAKTGEILATTQRPTFNADTK<br>EGITEDFVWRDILYQSNYEPGSGMKVMTLASSIDNNTFPSGEYFNSSEFKIADATTRDWDVNEGLTTGGMMTFLQGFAHSSNVGM<br>SLLEQKMGDATWLDYLKRFKFGVPTRFGLTDEYAGQLPADNIVSISQSSFGQGISVTQTQMLRAFTAIANDGVMLEPKFISAIYDTNN<br>QSVRKSQKEIVGNPVSKEAASTTRNHMILVGTDPLYGTMYNHYTGKPIITVPGQNVAVKSGTAQIADEKNGGYLVGSTNYIFSVVTM<br>NPAENPDFILYV
>112-like-2<br>GKDGIITYEKDRLGNIVPGTEQVSQQTVDGKDVYTTLSSPLQSFMETQMDAFLEKVKGKYMTATLVSAKTGEILATTQRPTFNADTK<br>EGITEDFVWRDILYQSNYEPGSGMKVMTLASSIDNNTFPSGEYFNSSEFKIADATTRDWDVNEGLTTGGMMTFLQGFAHSSNVGM<br>SLLEQKMGDATWLDYLKRFKFGVPTRFGLTDEYAGQLPADNIVSIAQSSFGQGISVTQTQMLRAFTAIANDGVMLEPKFISAIYDTNN<br>QSVRKSQKEIVGNPVSKEAASTTRNHMILVGTDPLYGTMYNHYTGKPIITVPGQNVAVKSGTAQIADEKNGGYLVGSTNYIFSVVTM<br>NPAENPDFILYV
>143-like<br>GTDGIITYEKDRLGNIVPGTEQVSQQTVDGKDVYTTLSSPLQSFMETQMDAFLEKVKGKYMTATLVSAKTGEILATTQRPTFNADTK<br>EGITEDFVWRDILYQSNYEPGSAMKVMTLASSIDNNTFPSGEYFNSSEFKIADATTRDWDVNDGLTTGGMMTFLQGFAHSSNVGM<br>SLLEQKMGDTTWLDYLNRFKFGVPTRFGLTDEYAGQLPADNIVSIAQSSFGQGISVTQTQMLRAFTAIANDGVMLEPKFISAIYDTNN<br>QSVRKSQKEIVGNPVSKEAASTTRNHMILVGTDPLYGTMYNHYTGKPIITVPGQNVAVKSGTAQIADEKNGGYLVGSTNYIFSVVTM<br>NPAENPDFILYV
>154-like<br>GKDGIITYEKDRLGNIVPGTEQVSQQTVDGKDVYTTISSTLQSFMETQMNAFQEKVKGKYMTATLVSAKTGEILATTQRPTFNADTK<br>EGITEDFVWRDILYQSNYEPGSAMKVMTLASSIDNNTFPSGEYFNSSEFKIADATTRDWDVNEGLTTGGMMTFLQGFAHSSNVGM<br>SLLEQKMGDATWLDYLKRFKFGVPTRFGLTDEYAGQLPADNIVSIAQSSFGQGISVTQTQMLRAFTAIANDGVMLEPKFISAIYDTNN<br>QSVRKSQKEIVGKPVSEDTASTTRNHMILVGTDPLYGTMYNHYTGKPIITVPGQNVAVKSGTAQIADEKNGGYLVGSTNYIFSAVTM<br>NPAENPDFILYV

Appendix 1 Table 7. Results of PCR amplification of antimicrobial resistance genes and Tn916-like transposon gene in 24 meropenem-nonsusceptible 15B/C-ST83 clinical isolate strains*

Isolate name	*ermB*	*tetM*	*cat*	*mef*(*A/E*)	*aphA3*	*int*-Tn916	*xis*-Tn916	*tnpR*-Tn916	*tnpA*-Tn916	Tn of Tn916-like	*folA* mutation†	*folP* insertion‡
B1136	+	+	+	–	–	+	+	–	–	Tn6002	+	+
B999-49	+	+	+	–	–	+	+	–	–	Tn6002	+	+
B1300936	+	+	+	–	–	+	+	–	–	Tn6002	+	+
1018-71	+	+	+	–	–	+	+	–	–	Tn6002	+	+
1025-08	+	+	+	–	–	+	+	–	–	Tn6002	+	+
1054-32	+	+	+	–	–	+	+	–	–	Tn6002	+	+
B1675	+	+	+	–	–	+	+	–	–	Tn6002	+	+
B0236	+	+	+	–	–	+	+	–	–	Tn6002	+	+
1148-64	+	+	+	–	–	+	+	–	–	Tn6002	+	+
1217-52	+	+	+	–	–	+	+	–	–	Tn6002	+	+
K54	+	+	+	–	–	+	+	–	–	Tn6002	+	+
K55	+	+	+	–	–	+	+	–	–	Tn6002	+	+
B2581	+	+	+	–	–	+	+	–	–	Tn6002	+	+
B5939	+	+	+	–	–	+	+	–	–	Tn6002	+	+
B4505	+	+	+	–	–	+	+	–	–	Tn6002	+	+
B1757	+	+	+	–	–	+	+	–	–	Tn6002	+	+
B3127	+	+	+	–	–	+	+	–	–	Tn6002	+	+
B2404	+	+	+	–	–	+	+	–	–	Tn6002	+	+
B8812	+	+	+	–	–	+	+	–	–	Tn6002	+	+
1244-61	+	+	+	–	–	+	+	–	–	Tn6002	+	+
1267-33	+	+	+	–	–	+	+	–	–	Tn6002	+	+
1276-72	+	+	+	–	–	+	+	–	–	Tn6002	+	+
K72	+	+	+	–	–	+	+	–	–	Tn6002	+	+
K110	+	+	+	–	–	+	+	–	–	Tn6002	+	+

*+, PCR positive; –, PCR negative.
†DNA sequence analysis revealed I100L substitution in *folA*.
‡DNA sequence analysis revealed insertion of one codon between bases 168 and 201 in *folP*.

Appendix 1 Table 8. Sequence coordinates of recombination sites

Number of recombination sites	Sequence coordinates	Length of recombination sites, bp	Genes or putative protein function	Recombination events
1	124732–124902	171	Part of *pspA* gene	15B/C-ST83
2	302151–329719	27,569	*cps* locus	15B/C-ST83
3	362492–389805	27,314	*tig*, *spi,* DNA helicase, *mutS2*, and *bacteriocin*	15B/C-ST83
4	750043–751380	1,338	IS861 of *orfA* and IS-Spn1 *orf8*	15B/C-ST83 and 23F-ST81
5	958399–961937	3,539	DNA primase, *ropD* and ABC transporter	15B/C-ST83 and 23F-ST81
6	1279909–1280068	160	Partial *tetM* in ICE element region	15B/C-ST83 and 23F-ST81
7	1487797–1526819	39,023	Autolysin, holing, putative phage protein cluster	15B/C-ST83 and 23F-ST81
8	1722313–1732499	10,187	Part of *psrP* gene	15B/C-ST83 and 23F-ST81
9	1831969–1839540	7,572	Siderophore uptake periplasmic binding protein, *rluB*, *scpB*, *scpA*, *xerD*, *glr*	15B/C-ST83 and 23F-ST81
10	1938458–1941716	3,259	*pflC*, *purR*	15B/C-ST83 and 23F-ST81
11	2094386–2095149	764	Putative DNA binding protein	15B/C-ST83
12	2171700–2171829	130	Part of *cbpA* gene	15B/C-ST83

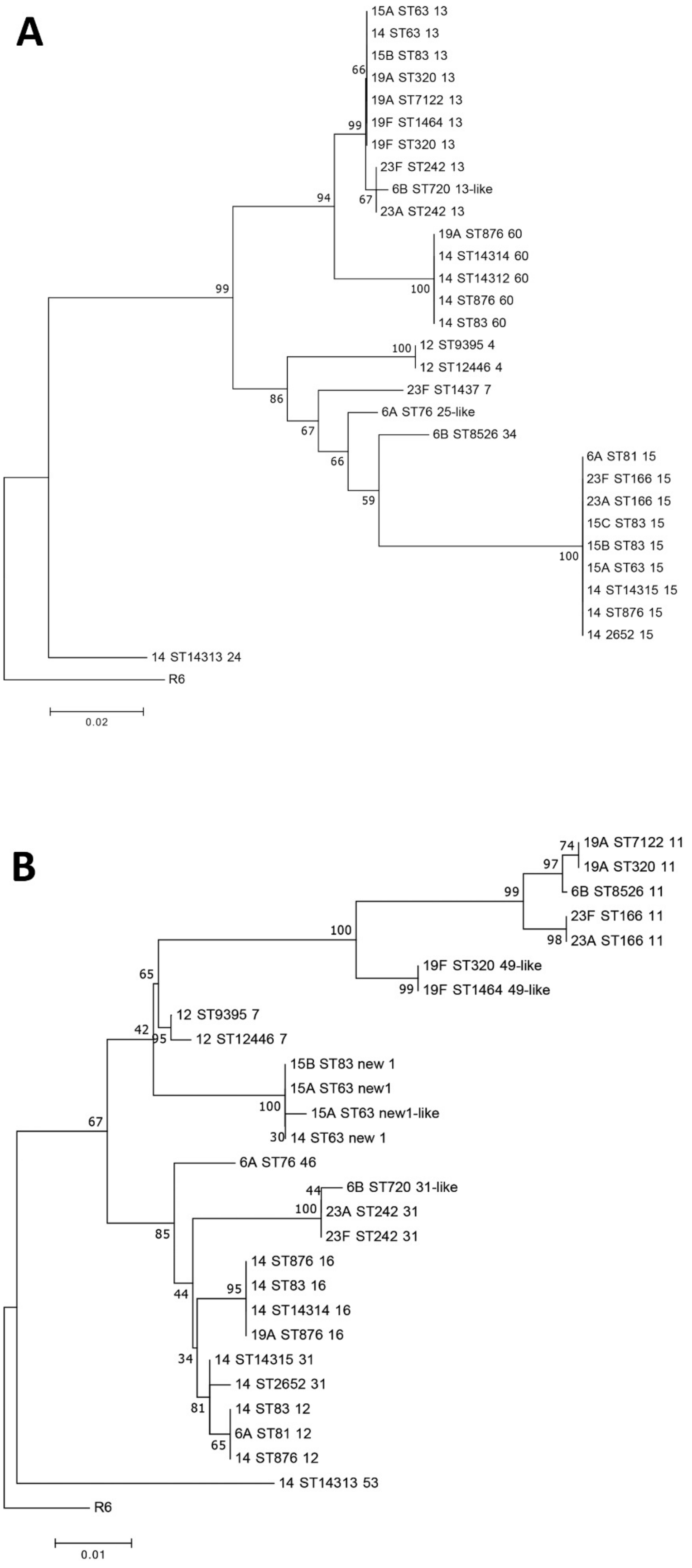
A
15A ST63 13
14 ST63 13
15B ST83 13
19A ST320 13
19A ST7122 13
19F ST1464 13
19F ST320 13
23F ST242 13
6B ST720 13-like
23A ST242 13
19A ST876 60
14 ST14314 60
14 ST14312 60
14 ST876 60
14 ST83 60
12 ST9395 4
12 ST12446 4
23F ST1437 7
6A ST76 25-like
6B ST8526 34
6A ST81 15
23F ST166 15
23A ST166 15
15C ST83 15
15B ST83 15
15A ST63 15
14 ST14315 15
14 ST876 15
14 2652 15
14 ST14313 24
R6
66
99
67
94
100
99
100
86
67
66
59
100
0.02
B
19A ST7122 11
19A ST320 11
6B ST8526 11
23F ST166 11
23A ST166 11
19F ST320 49-like
19F ST1464 49-like
12 ST9395 7
12 ST12446 7
15B ST83 new 1
15A ST63 new1
15A ST63 new1-like
14 ST63 new 1
6A ST76 46
6B ST720 31-like
23A ST242 31
23F ST242 31
14 ST876 16
14 ST83 16
14 ST14314 16
19A ST876 16
14 ST14315 31
14 ST2652 31
14 ST83 12
6A ST81 12
14 ST876 12
14 ST14313 53
R6
74
97
99
98
100
99
65
42
95
100
30
67
44
100
85
95
44
34
81
65
0.01

C

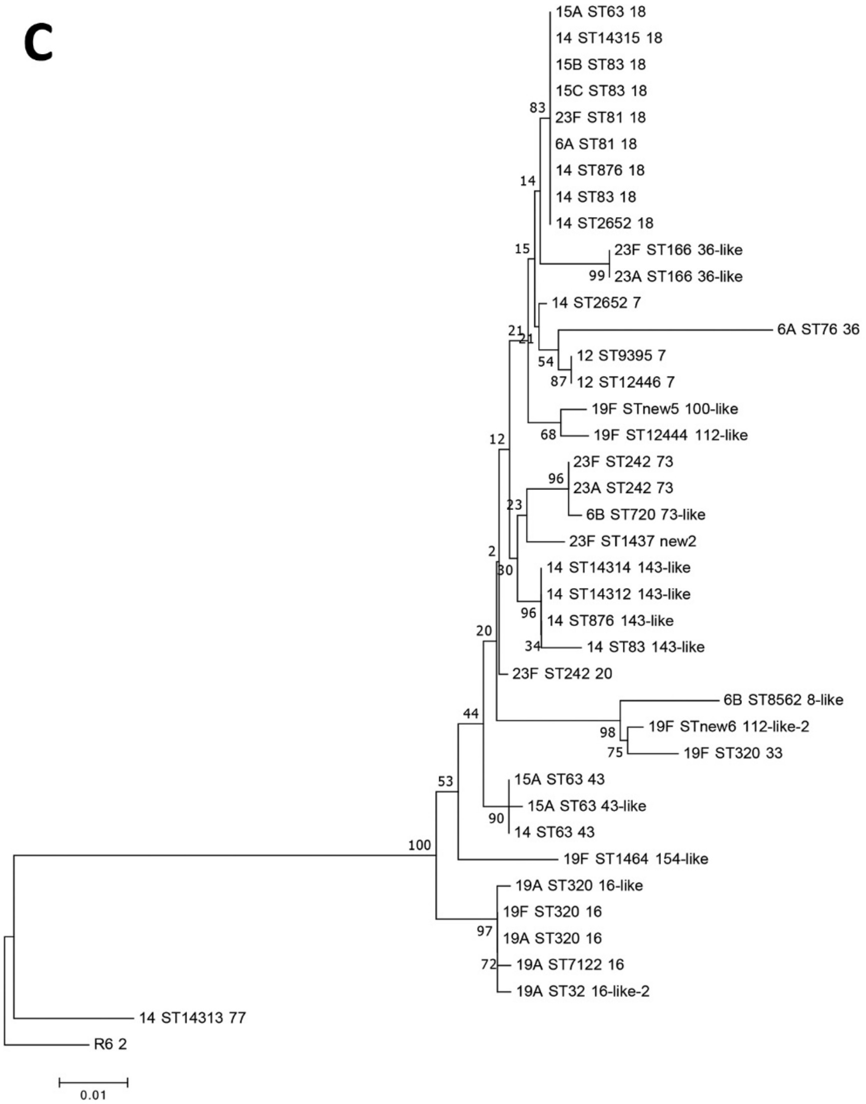

Appendix 1 Figure 1. Neighbor-joining trees showing the relatedness of the amino acid sequence of A) PBP1a; B) PBP2b; and C) PBP2x of *Streptococcus pneumoniae*, Taiwan, 2013–2017. The amino acid sequence of PBP in the *S. pneumoniae* R6 strain is included as a reference. The evolutionary history was inferred using the neighbor-joining method (*6*). The optimal trees had branch length sums of A) 0.29717399; B) 0.21161535; and C) 0.27634923. The percentage of replicate trees in which the associated taxa clustered together in the bootstrap test (1,000 replicates) are shown next to the branches (*7*). The evolutionary distances were computed by using the Poisson correction method (*8*). Scale bars indicate the number of amino acid substitutions per site. PBP, penicillin binding protein.

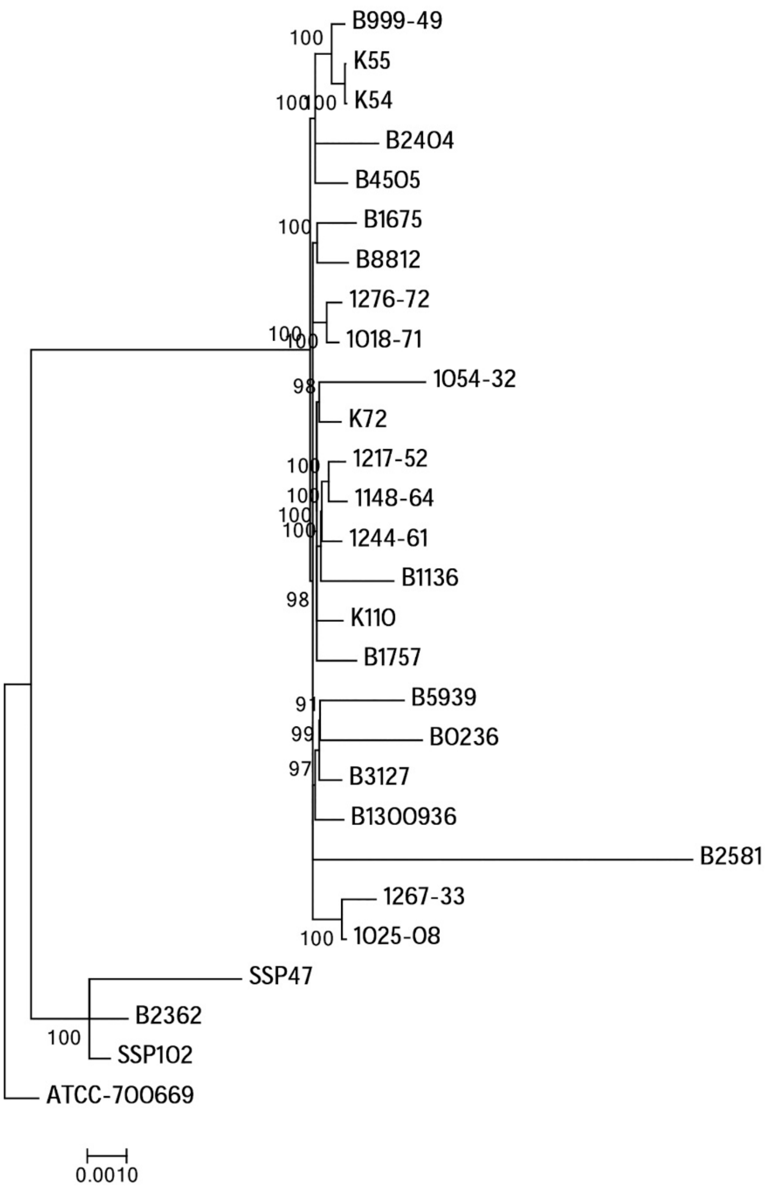

Appendix 1 Figure 2. Recombination-free phylogenetic tree of draft genomes of *Streptococcus pneumoniae* isolates collected in Taiwan during 2013–2017. The tree was created by using the maximum likelihood method in RAxML (https://github.com/stamatak/standard-RAxML) and the GTRCAT model with 1,000 bootstrap values in Recombination Detection Program (RDP) version 4.89 (*9*). Draft genomes were generated from contigs by using a post-assembly genome-improvement toolkit (*10*), including 24 meropenem-nonsusceptible serotype 15B/C-ST83, and 3 23F-ST81 strains. The PMEN1 reference strain, accession no. ATCC-700669, was added as an outgroup. Parsnp version 1.2 of the Harvest Suite (*11*) was applied to perform a core alignment of these genomes before constructing the tree using RDP. Scale bar represents nucleotide substitutions per site.

References

1. Sanbongi Y, Ida T, Ishikawa M, Osaki Y, Kataoka H, Suzuki T, et al. Complete sequences of six penicillin-binding protein genes from 40 *Streptococcus pneumoniae* clinical isolates collected in Japan. Antimicrob Agents Chemother. 2004;48:2244–50. PubMed https://doi.org/10.1128/AAC.48.6.2244-2250.2004

2. Talebi M, Azadegan A, Sadeghi J, Ahmadi A, Ghanei M, Katouli M, et al. Determination of characteristics of erythromycin resistant *Streptococcus pneumoniae* with preferred PCV usage in Iran. PLoS One. 2016;11:e0167803. PubMed https://doi.org/10.1371/journal.pone.0167803

3. Setchanova L, Tomasz A. Molecular characterization of penicillin-resistant *Streptococcus pneumoniae* isolates from Bulgaria. J Clin Microbiol. 1999;37:638–48. PubMed https://doi.org/10.1128/JCM.37.3.638-648.1999

4. Lattar SM, Wu X, Brophy J, Sakai F, Klugman KP, Vidal JE. A mechanism of unidirectional transformation, leading to antibiotic resistance, occurs within nasopharyngeal pneumococcal biofilm consortia. MBio. 2018;9:e00561–18. PubMed https://doi.org/10.1128/mBio.00561-18

5. Haasum Y, Ström K, Wehelie R, Luna V, Roberts MC, Maskell JP, et al. Amino acid repetitions in the dihydropteroate synthase of *Streptococcus pneumoniae* lead to sulfonamide resistance with limited effects on substrate $K_{(m)}$. Antimicrob Agents Chemother. 2001;45:805–9. PubMed https://doi.org/10.1128/AAC.45.3.805-809.2001

6. Saitou N, Nei M. The neighbor-joining method: a new method for reconstructing phylogenetic trees. Mol Biol Evol. 1987;4:406–25. PubMed

7. Felsenstein J. Confidence limits on phylogenies: an approach using the bootstrap. Evolution. 1985;39:783–91. PubMed https://doi.org/10.1111/j.1558-5646.1985.tb00420.x

8. Zuckerkandl EPL. Evolutionary divergence and convergence in proteins. In: Bryson V and Vogel HJ, editors. Evolving genes and proteins. New York: Academic Press. 1965:97–166.

9. Martin DP, Murrell B, Golden M, Khoosal A, Muhire B. RDP4: detection and analysis of recombination patterns in virus genomes. Virus Evol. 2015;1:vev003. PubMed https://doi.org/10.1093/ve/vev003

10. Swain MT, Tsai IJ, Assefa SA, Newbold C, Berriman M, Otto TD. A post-assembly genome-improvement toolkit (PAGIT) to obtain annotated genomes from contigs. Nat Protoc. 2012;7:1260–84. PubMed https://doi.org/10.1038/nprot.2012.068

11. Treangen TJ, Ondov BD, Koren S, Phillippy AM. The Harvest suite for rapid core-genome alignment and visualization of thousands of intraspecific microbial genomes. Genome Biol. 2014;15:524. PubMed https://doi.org/10.1186/s13059-014-0524-x
